# An exploratory study investigating the effect of foot type and foot orthoses on gluteus medius muscle activity

**DOI:** 10.1186/s12891-020-03683-7

**Published:** 2020-10-07

**Authors:** Sean Sadler, Martin Spink, Xanne Janse de Jonge, Vivienne Chuter

**Affiliations:** 1 Discipline of Podiatry, University of Newcastle, Ourimbah, NSW 2258 Australia; 2 Priority Research Centre for Physical Activity and Nutrition, University of Newcastle, Newcastle, NSW 2308 Australia; 3 Discipline of Exercise and Sport Science, University of Newcastle, Ourimbah, NSW 2258 Australia

**Keywords:** Gluteus medius, Foot type, Prefabricated foot orthoses, Electromyography

## Abstract

**Background:**

Abnormal gluteus medius muscle activity is associated with a number of musculoskeletal conditions. Research investigating the effect of foot type and foot orthoses on gluteus medius muscle activity is both conflicting and limited. The primary aim was to investigate the relationship between foot type and gluteus medius muscle activity during shod walking. The secondary aims of this study were to explore the effect and amount of usage of a pair of unmodified prefabricated foot orthoses on gluteus medius muscle activity during shod walking.

**Methods:**

Foot type was determined using the foot posture index and gluteus medius muscle activity was measured with surface electromyography in 50 healthy adults during shod walking. Participants were then fitted with prefabricated foot orthoses and required to return after 4 weeks. Pearson’s correlation and one-way ANOVA were used to determine effect of foot type. Paired t-tests and ANCOVA were used to determine effect of foot orthoses.

**Results:**

Participants with a cavus foot type demonstrated significantly more gluteus medius mean (*p* = 0.04) and peak amplitude (*p* = 0.01), and a greater range in amplitude (*p* = 0.01) compared to participants with a neutral foot type. Compared to a planus foot type, participants with a cavus foot type demonstrated significantly larger mean (*p* = 0.02) and peak amplitude (*p* = 0.01), and a greater range in amplitude (*p* = 0.01). Prefabricated foot orthoses did not change the gluteus medius muscle activity.

**Conclusion:**

When assessing healthy adults with a cavus foot type, clinicians and researchers should be aware that these participants may display higher levels of gluteus medius muscle activity during gait compared to neutral and planus type feet. Additionally, clinicians and researchers should be aware that the type of prefabricated foot orthoses used did not change gluteus medius muscle activity over 4 weeks. Future research should aim to explore this relationship between foot type and gluteus medius muscle activity in larger sample sizes, consider the potential role of other lower extremity muscles and biomechanical variables, and investigate if these findings also occur in people with pathology.

## Introduction

Gluteus medius muscle activity plays a significant role in controlling motion of the lumbopelvic-hip complex and lower extremity during gait [1]. Dysfunction of the gluteus medius muscle has been linked with both reduced frontal and transverse plane control of the pelvis and femur [2]. These changes have been shown to manifest in a dynamic knee valgus, increased anterior pelvic tilt, and greater lumbar lordosis, all of which have been linked to musculoskeletal pathologies including patellofemoral pain syndrome (PFPS) [3, 4] and low back pain [5]. More distally, weakness of the hip muscles, resulting in a Trendelenburg gait pattern [6], as well as altered activation patterns have been implicated in the presence of dynamic excessive foot pronation [3, 7, 8]. This loss of pelvic and femoral control can lead to internal lower limb rotation, and through the tibio-calcaneal coupling mechanism, result in increased subtalar joint pronation and a dynamic planus foot type [9]. Furthermore, there is evidence of targeted hip muscle strengthening altering dynamic rearfoot motion, suggesting a possible interrelationship [10].

Previous research has demonstrated that the increased motion associated with a planus foot type is associated with increased invertor and decreased evertor lower leg muscle activity [11]. This relationship between foot type and range of motion is also suggested to influence muscle activity of more proximal muscles such as the gluteus medius [5, 12, 13]. This is supported by evidence of increased gluteus medius muscle activity in people with chronic nonspecific low back pain and excessively pronated feet [5]. In people with cavus type feet, the lack of motion has been shown to result in increased lower leg muscle activity compared to planus and neutral foot types [14]. This study also found that responses between foot type groups during barefoot walking in female adults was subject specific and may be localised to the lower limb. Therefore, the effect that cavus feet have on muscles in the lumbo-pelvic hip complex, such as the gluteus medius, remains underexplored.

Supporting the relationship between foot function and movement at the hip, foot orthoses are used to treat a range of musculoskeletal pathologies including those associated with gluteus medius weakness, such as PFPS [11, 15, 16]. Although foot orthoses are used to treat a range of musculoskeletal conditions associated with different foot types, their mechanism of action is unclear [16–18]. One theory suggests that foot orthoses apply a sensory input through the sole of the foot to improve muscular efficiency while reducing fatigue of muscles throughout the lower limb and pelvis [11, 16, 19–21]. However, several studies have reported no effect of foot orthoses on parameters of gluteus medius function during functional tasks (i.e. step-ups) [22] and gait immediately following application, and over time periods of up to 4 weeks in healthy individuals [20]. In one study, that recruited 15 healthy adults with cavus feet, custom made foot orthoses did not affect gluteus medius muscle activity over 4 weeks [23]. Conversely, immediately after application of prefabricated foot orthoses or foot wedges, gluteus medius activity has been shown to be increased in healthy participants during a single leg squat task in people with planus, neutral, and cavus foot types [24], but reduced during a step-up task in those with PFPS [25] and have a delayed onset during walking gait [12]. These differences highlight that foot orthoses can effect gluteus medius muscle activity, with a previous study suggesting that foot orthoses increase sensory feedback through increased contact, such as in those with a cavus foot type [24]. Furthermore, the relatively small sample sizes, differences in orthotic designs and length of follow-up, the large variance in study populations and testing methods, as well as most studies not considering foot type, may all partly explain these inconsistent findings and suggest that further research is needed.

Determining if there is an effect of foot type (cavus, neutral, and planus) on gluteus medius function and if this is altered by foot orthoses is a key element in understanding the role of foot orthoses in the treatment of musculoskeletal injuries of the lower limb. Consequently, investigating the effect of unmodified prefabricated foot orthoses on a range of gluteus medius muscle activity measures during gait after a short period of acclimatisation, and in a larger healthy population, will help clarify if there is a consistent effect of foot orthoses more proximally.

The primary aim of this exploratory study was to investigate the relationship between foot type and gluteus medius muscle activity during gait. The secondary aims were to investigate the effect that a pair of unmodified prefabricated foot orthoses has on gluteus medius muscle activity over 4 weeks, and to determine if this was affected by the amount of usage.

## Methods

### Participants

Ethics approval was granted by the University of Newcastle Human Research Ethics Committee (H-2017-0345) and written informed consent was obtained from all participants. A convenience sample of healthy participants was recruited from the Central Coast community and the University of Newcastle podiatry teaching clinic at Wyong Hospital, both located in New South Wales, Australia. Inclusion criteria were adults aged 18 to 65 years. Exclusion criteria were use of any foot orthoses in the past 12 months, previous lower back surgery, pregnancy, any type of low back pain within the past 12 months, or any inflammatory (e.g. rheumatoid arthritis), or neurological conditions. Additionally, participants with an allergy to silver, any electronic implants (e.g. pace maker), or any wounds on the outer part of their right hip were also ineligible due to test protocol requirements.

### Procedures

Data were collected at the University of Newcastle podiatry clinic at Wyong Hospital or the Ourimbah campus. Physical activity level was measured with the short version of the International Physical Activity Questionnaire (IPAQ-7) [26]. Participants’ activity level was categorised as low, moderate, or high based on the total volume and number of days they perform each level of activity.

For all lower limb measurements, only the right limb was used to adhere to the assumption of independence of data [27]. The foot posture index (FPI) was used to determine foot type [28]. In this study, we classified foot type as cavus (FPI < 0), neutral (FPI 0–5), or planus (FPI > 5) [29]. The FPI has shown good validity [30] and moderate to high inter-rater and test retest reliability [31].

Gluteus medius muscle activity was measured using surface EMG (Delsys Trigno™ wireless system, Natick, Mass., USA). The sampling frequency was 2 kHz with an amplification gain of 1000. Raw EMG was band pass filtered between 20 and 450 Hz using a fourth order Butterworth filter. Skin was lightly abraded and cleaned with an alcohol wipe, and when necessary, hair removed. Following this, a single rectangular Delsys Trigno™ EMG sensor (27 mm × 37 mm × 15 mm), with 99% silver electrode contacts, was adhered to the skin overlying the right gluteus medius muscle, approximately half way between the line from the iliac crest and the greater trochanter, and parallel to the direction of the muscle fibres [32]. Maximal voluntary isometric contraction (MVIC) of the gluteus medius muscle was measured during the side-lying hip abduction test. Participants were instructed to abduct their hip to neutral and then continue abducting whilst the leg was held in stationary position by the examiner who applied resistance to the right ankle. Three MVIC, each held for 5 s, and with 60 s rest between each contraction were measured [33]. Subsequent dynamic gluteus medius muscle activity was expressed as a percentage of the highest MVIC to allow for a comparison between participants. Surface EMG is considered a reliable tool for measuring gluteus medius muscle activity and activation [34].

Foot switches (Delsys FSR sensors, Natick, Mass., USA) were fitted to the plantar aspect of the right heel and the interphalangeal joint of the right hallux to record heel strike and toe off respectively. Gluteus medius muscle activity was measured shod on a hard level surface at a self-selected speed for approximately 10 s with a minimum of seven strides [35]. Participants wore the same self-selected lace-up enclosed shoes, that were suitable to have an orthotic device fitted, at each of the data collection sessions.

**Fig. 1:**
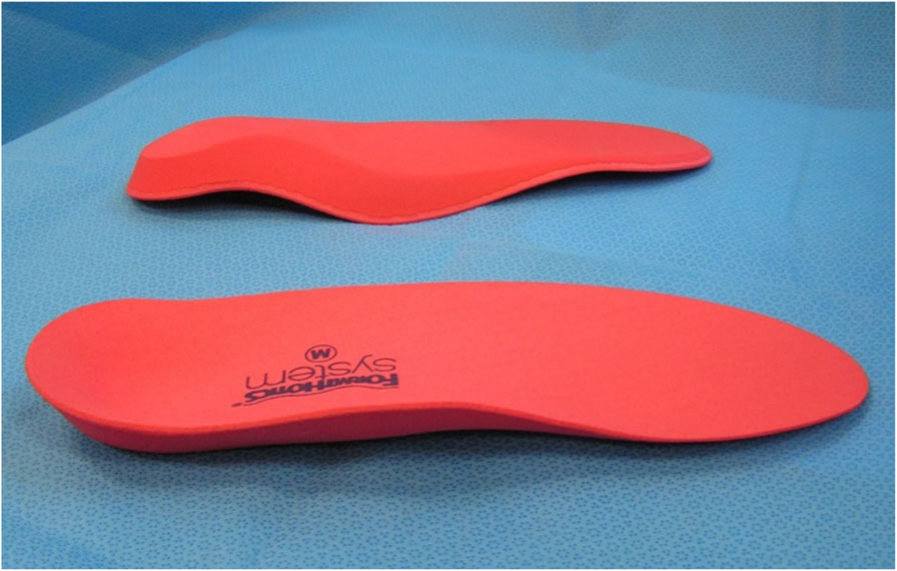
Prefabricated orthoses (Formthotics™, Foot Science International Ltd., Christchurch, New Zealand) used in the study

Participants were fitted with a pair, one for each foot, of unmodified full-length Formthotics™ prefabricated foot orthoses (Foot Science International Ltd., Christchurch, New Zealand) (Fig. 1) by a podiatrist (SS) with 5 years clinical experience. The foot orthoses are a dual density device consisting of a firm density base (density rating of 160 kg/m3) and a soft density top cover (density rating of 70 kg/m3). This device was chosen because previous research has shown that Formthotics™ prefabricated foor orthoses can influence lower leg muscle activity [36, 37], plus this device allows for it to be heat moulded to the participant’s foot as per the manufacturer’s instructions resulting in a closer fit to the morphology of the foot regardless of foot type. The size of the full-length Formthotics™ device closest to the participant’s shoe size was selected, with the forefoot region trimmed with scissors as needed to fit into their shoes. The insert that was part of the participant’s shoes was removed before fitting. Participants were instructed to gradually wear the device in, using comfort levels as a guide, and aim to wear them as much as possible. Participants were also provided with a paper-based diary to record the number of hours per day that they wore the orthoses. To aid completeness of reporting, we have used the template for intervention description and replication (TIDieR) checklist [38].

At the follow-up session approximately 4 weeks after baseline testing, only gluteus medius muscle activity (MVIC and during shod walking with orthoses) was measured. At this session, the assessor followed exactly the same methods used at baseline for sensor placement and measurement of muscle activity, including the order of testing. Additionally, participants were provided with the same instructions for assessment.

### Outcomes

The primary outcome was the effect of foot posture on baseline gluteus medius muscle activity. Secondary outcomes were the change in gluteus medius muscle activity during stance phase after 4 weeks of orthotic wear, and the effect of the amount of orthotic usage on this change. EMG variables recorded were mean amplitude, peak amplitude, minimum amplitude, and peak to peak (range).

### Statistical analysis

Gluteus medius EMG data were prepared and analysed using Delsys analysis software (EMGworks). The remove mean script was applied to raw data followed by the amplitude script, which included performing the root mean square over a 125 ms window and simultaneously normalising it against the maximum values from the three MVICs, to express dynamic gluteus medius muscle activity as a percentage of MVIC. EMG and foot switch data for the first two gait cycles were excluded and the next 5 cycles were used in the cyclical analysis script to get a time normalised average cycle.

Statistical analyses were performed using SPSS (version 25.0 Chicago, Illinois, USA). Means and standard deviations were calculated for demographic, anthropometric, FPI, and EMG variables. Pearson’s correlation and one-way ANOVA were used to explore the relationship between foot type and all baseline EMG variables. The Rasch foot type values were used so the FPI data could be treated as a continuous variable [30]. The strength of the correlation was interpreted as small (r = 0.10–0.29), moderate (r = 0.30–0.49), and large (r = 0.50–1.0) [39]. For one-way ANOVA, the partial eta squared effect size was used and interpreted as small (0.01–0.05), medium (0.06–0.13), and large (≥0.14) [39]. Post-hoc comparisons using the Tukey HSD tests were planned to investigate differences in EMG variables between foot type groups. Paired t-tests were performed to investigate the effect of the prefabricated foot orthoses on gluteus medius muscle activity over 4 weeks. Analysis of covariance (ANCOVA) was used to assess the effect of prefabricated foot orthotic usage on each EMG variable. The covariate for each analysis was the baseline EMG variable. The grouping variable was orthotic usage, with the median hours of usage used to split the group in two. Assumptions for all analyses were met.

## Results

The majority of participants were moderately to highly active, either in the normal or overweight categories for body mass index, and most had a neutral or planus foot type (Table 1). All 50 participants included at baseline returned for follow-up testing.

**Table 1:** Participant characteristics

Age, mean years (SD)	34.34 (13.47)
Female, n (% of total)	26 (52)
BMI, mean Kg/m^2^ (SD)	25.07 (4.06)
Waist circumference, mean cm (SD)	81.11 (11.84)
Follow-up time, mean days (range)	27.90 (25–30)
Smoking status	
Previous, n (% of total)	10 (20)
Current, n (% of total)	5 (10)
Never, n (% of total)	35 (70)
Physical activity level (IPAQ-7)	
Low, n (% of total)	9 (18)
Moderate, n (% of total)	15 (30)
High, n (% of total)	26 (52)
Foot type	
Cavus FPI < 0, n (% of total), mean (range)	10 (20), −1.6 (−1 to −5)
Neutral FPI 0–5, n (% of total), mean (range)	24 (48), 3.14 (1 to 5)
Planus FPI > 5, n (% of total), mean (range)	16 (32), 6.47 (6 to 9)
Orthotic usage	
Mean hours (SD)	107.83 (64.05)
Median hours (IQR)	112.75 (101.25)
Range hours	9–242.5

*SD* Standard deviation, *BMI* Body mass index, *IPAQ-7* Short version of the international physical activity questionnaire, *FPI* Foot posture index, *IQR* Interquartile range

The relationship between FPI and each of the baseline EMG variables was investigated using Pearson’s correlations (Table 2). For mean amplitude, peak amplitude, and peak to peak amplitude there was a medium, significant, negative correlation between FPI and each of these variables at baseline. This means that the lower the FPI score (more cavus), the higher the baseline gluteus medius mean and peak amplitude, and the greater the range in muscle activity.

**Table 2:** Pearson correlation between Rasch foot posture index and baseline electromyographic variables

	Mean amplitude	Peak amplitude	Minimum amplitude	Peak to peak amplitude
Rasch foot posture index	−0.348^a^	−0.372^b^	−0.158	−0.367^b^

^a^Correlation is significant at the 0.05 level (2-tailed)

^b^Correlation is significant at the 0.01 level (2-tailed)

At baseline during shod gait there was a statistically significant difference between foot type groups for mean amplitude, F (2, 47) = 4.39, *p* = 0.02, peak amplitude, F (2, 47) = 5.27, *p* = 0.01, and peak to peak amplitude, F (2, 47) = 4.91, *p* = 0.01. The effect sizes for mean, peak, and peak to peak amplitude were 0.16, 0.18, and 0.17 respectively, all of which are considered large.

Post-hoc comparisons using the Tukey HSD tests indicated that those participants with a cavus foot type demonstrated a statistically significant increase in gluteus medius mean and peak amplitude, and a greater range in amplitude, compared to neutral and planus foot types (Table 3).

**Table 3:** One-way ANOVA for baseline EMG variables and foot type

	Mean %MVIC (SD)	Mean difference (95%CI)	*P* value
Mean amplitude			
cavus vs neutral*	14.32 (8.88) vs 8.18 (6.63)	6.13 (0.34 to 11.93)	0.04
cavus vs planus*	14.32 (8.88) vs 7.12 (3.47)	7.20 (0.99 to 13.41)	0.02
neutral vs planus	8.18 (6.63) vs 7.12 (3.47)	1.06 (−3.91 to 6.03)	0.86
Peak amplitude			
cavus vs neutral*	29.88 (17.51 vs 16.09 (11.89)	13.79 (2.65 to 24.93)	0.01
cavus vs planus*	29.88 (17.51) vs 15.51 (8.23)	14.37 (2.44 to 26.30)	0.02
neutral vs planus	16.09 (11.89) vs 15.51 (8.23)	0.58 (−8.97 to 10.13)	0.99
Peak to peak amplitude			
cavus vs neutral*	26.79 (16.85) vs 13.94 (11.00)	12.85 (2.28 to 23.43)	0.01
cavus vs planus*	26.79 (16.85) vs 13.91 (8.15)	12.89 (1.56 to 24.21)	0.02
neutral vs planus	13.94 (11.00) vs 13.91 (8.15)	0.03 (−9.04 to 9.10)	1.00

*indicates statistical significance at *p <* 0.05

*%MVIC* Percentage of maximum voluntary isometric contraction, *SD* Standard deviation, *95%CI* 95% confidence interval

A paired-samples t-test found no statistically significant effect of prefabricated foot orthoses on gluteus medius muscle activity over 4 weeks (Table 4).

**Table 4:** Electromyographic (EMG) values. All values are % of MVIC

EMG variable	Baseline (*n* = 50)			Follow-up (*n* = 50)			Mean Change (95% CI)	*P* value
	mean	SD	95% CI	mean	SD	95%CI		
Mean amplitude	9.07	6.79	7.14 to 11.00	9.06	5.56	7.48 to 10.64	−0.01 (−1.63 to 1.62)	0.99
Peak amplitude	18.67	13.25	14.90 to 22.43	19.04	12.87	15.38 to 22.69	0.37 (−2.67 to 3.41)	0.81
Minimum amplitude	2.17	2.07	1.58 to 2.76	2.42	1.73	1.92 to 2.91	0.25 (−0.33 to 0.82)	0.39
Peak to peak amplitude	16.50	12.51	12.94 to 20.05	16.62	12.52	13.06 to 20.18	0.12 (−2.68 to 2.93)	0.93

*MVIC* Maximum voluntary isometric contraction, *SD* Standard deviation, *95%CI* 95% confidence interval

Separate one-way between groups analyses of covariance (ANCOVA) found that the amount of orthotic usage did not make a statistically significant difference in any of the EMG variables at follow-up (Table 5).

**Table 5:** Electromyographic (EMG) variables by orthotic usage. All values are % of MVIC. Values are means (standard deviations) unless otherwise stated

EMG variable	< 112 h orthotic usage (*n* = 25)		≥112 h orthotic usage (*n* = 25)		Adjusted mean difference (95% CI)	*P* value
	Baseline	Follow-up	Baseline	Follow-up		
Mean amplitude	11.31 (8.41)	9.28 (6.55)	6.83 (3.59)	8.84 (4.92)	1.93 (−0.79 to 4.64)	0.16
Peak amplitude	23.06 (16.23)	20.06 (16.10)	14.27 (7.40)	18.01 (8.76)	4.08 (−1.72 to 9.89)	0.16
Minimum amplitude	2.68 (2.63)	2.37 (1.97)	1.66 (1.16)	2.46 (1.50)	0.50 (−0.42 to 1.42)	0.28
Peak to peak amplitude	20.38 (15.45)	17.69 (15.75)	12.61 (7.00)	15.54 (8.34)	3.57 (−1.88 to 9.02)	0.19

*MVIC* Maximum voluntary isometric contraction, *95%CI* 95% confidence interval

## Discussion

The primary aim of this study was to explore the relationship between foot type and gluteus medius muscle activity. We found a statistically significant, moderate negative correlation between foot type and baseline gluteus medius mean, peak, and peak to peak amplitude. This means that those with a lower foot posture index score (i.e. cavus foot type) have higher levels of gluteus medius muscle activity. Further analysis demonstrated that the significant difference occurred between cavus and neutral, as well as, cavus and planus foot types, while no significant difference in gluteus medius muscle activity between neutral and planus foot types was found.

To the authors’ knowledge, this is the first study to investigate the effect of a cavus foot type compared to neutral and planus foot types on gluteus medius muscle activity in shod walking in healthy participants. The increased mean and peak, and a greater range in gluteus medius muscle activity in cavus feet compared to neutral and slightly pronated foot types demonstrated in this study is supported by previous research that has found both higher levels of peroneus longus muscle activity [14] and earlier onset of the vastus lateralis muscle [40] in people with cavus feet. These findings suggest that the lack of motion demonstrated in cavus feet may result in muscles on the lateral aspect of the lower limb and pelvis to increase their level of activity and activate earlier to help compensate for the increased vertical loading rate demonstrated in people with cavus type feet [40, 41].

Our findings of lower gluteus medius mean and peak amplitude in participants with planus foot types are in contrast to previous research that has shown higher levels of activity in people with chronic low back pain and pronated feet [5]. However, a recent systematic review found the relationship between low back pain and gluteus medius muscle activity to be inconclusive [42]. Another systematic review investigating the effect of foot posture on lower leg muscle activity during gait found some evidence to show that in people with planus type feet, greater levels of anti-pronator muscle activity (e.g. tibilias posterior and tibialis anterior) and reduced levels in evertor muscle activity occur compared to neutral and cavus feet [11]. Given the proposed role of the gluteus medius muscle in controlling pelvic and lower extremity motion during gait, perhaps the propagation of motion distally, resulting in increased foot motion, is attempted to be controlled by more closely located muscles, such as those muscles in the lower leg. Whether this relationship, or variables such as gluteus medius muscle strength, kinetic, or kinematic differences between foot type groups are responsible for these findings is not clear and requires additional investigation.

The secondary aims of this study were to explore the effect and amount of usage of a pair of unmodified prefabricated foot orthoses on gluteus medius muscle activity during shod walking. We found that the pair of unmodified prefabricated foot orthoses, irrespective of their amount of use over 4 weeks, did not change the gluteus medius EMG variables measured. This finding is in agreement with previous studies investigating the effect of custom made polypropylene foot orthoses on gluteus medius muscle activity over 4 weeks in healthy participants [20] and those with cavus type feet [23]. This suggests that both firm custom made and the softer prefabricated foot orthoses heat moulded to the participant’s foot used in the current study are unlikely to provide significant changes in gluteus medius muscle activity during gait in healthy people.

The current trial did not investigate the immediate effects of prefabricated foot orthoses on gluteus medius muscle activity in healthy participants during functional tasks. However, previous studies in this field have shown mixed results. A study of 30 participants, 10 with each foot type, tested each participant in a single leg squat in four conditions of a prefabricated orthotic device with a medial rearfoot post, a lateral rearfoot post, a neutral rearfoot post, and no orthoses. They found an increase in gluteus medius muscle activity in all three orthotic conditions compared to no orthoses regardless of foot type [24]. These changes were not replicated in a similarly posted prefabricated device in healthy participants during a step-up task [22]. However, these devices were able to decrease peak gluteus medius amplitude in those with PFPS [25]. The effect of foot orthoses on gluteus medius muscle activity immediately after their introduction might occur due to potential instability introduced by the device, creating small kinematic or kinetic changes which are attempted to be controlled by increasing muscle activity. However, our findings suggest that any possible effect of the pair of prefabricated foot orthotic devices used in this study have on gluteus medius muscle activity is reduced after a period of acclimatisation (4 weeks).

This study has a number of limitations. We were unable to calculate a sample size a priori, however the results of this study can be used to calculate sample sizes for future research in this area. The relatively small sample size within each of the foot type groups, the absence of pathology, and low BMI and moderate to high levels of physical activity of the included participants all limit the generalisability of these findings. Although the same methods for measurement of muscle activity were identical for each session, there is the possibility that differences in EMG sensor placement, or the self-selected speed at which participants walked was slightly different. While all shoes were appropriate to have the pair of unmodified prefabricated foot orthoses fitted, perhaps some differences in shoe design could have influenced the findings. There is some evidence demonstrating an association between the FPI and dynamic foot function [43–45]. However, due to this relationship not being strong for all measures of dynamic foot function and because we collapsed FPI data into foot type categories, some caution is advised when interpreting the relationship found between foot type and gluteus medius muscle activity. Additionally, the measurement of a number of other muscles’ activity level, as well as, foot and lower extremity biomechanical differences that might exist between foot types, were not measured which could have accounted for the findings.

## Conclusion

We found that people with a cavus foot type demonstrated increased levels of gluteus medius muscle activity compared to neutral and planus type feet during a short period of level ground shod walking. The pair of unmodified prefabricated foot orthoses did not significantly change gluteus medius muscle activity over a 4 week period, regardless of the amount of usage. Clinicians and researchers should be aware that participants with cavus feet may display higher levels of gluteus medius muscle activity during gait compared to neutral and planus type feet. Future research should aim to explore this relationship between foot type and gluteus medius muscle activity in larger sample sizes, consider the potential role of other lower extremity muscles, and investigate if these findings also occur in people with pathology.

## Data Availability

The datasets used and/or analysed during the current study are available from the corresponding author on reasonable request.

## Abbreviations

SD: Standard deviation
BMI: Body mass index
IPAQ-7: Short version of the international physical activity questionnaire
FPI: Foot posture index
IQR: Interquartile range
%MVIC: Percentage of maximum voluntary isometric contraction
CIs: 95% confidence interval
